# Supplementary material for: Ratiometric Matryoshka biosensors from a nested cassette of green- and orange-emitting fluorescent proteins
Source: Nat Commun. 2017 Sep 5;8:431. doi: 10.1038/s41467-017-00400-2 (PMC5585204; doi:10.1038/s41467-017-00400-2)
Supplement: Supplementary file 1 — Supplementary Information [file 41467_2017_400_MOESM1_ESM.pdf]

Description of Supplementary Files

File Name: Supplementary Information
Description: Supplementary Figures, Supplementary Tables, Supplementary Notes and Supplementary References.

File Name: Supplementary Movie 1
Description: Average z-stack projection of emission in the green channel (500-540 nm). Lookup table is shown in lower left in arbitrary units.

File Name: Supplementary Movie 2
Description: Average z-stack projection of emission in the orange channel (570-650 nm). Lookup table is shown in lower left in arbitrary units.

File Name: Supplementary Movie 3
Description: Ratio of green-to-orange emission in average z-stack projections after application of a binary mask, shown in SM5. Lookup table is shown in lower left corner, with ratio values listed.

File Name: Supplementary Movie 4
Description: Transmitted light (Brightfield), single-plane only.

File Name: Supplementary Movie 5
Description: Binary mask generated from projection of orange channel and applied to ratiometric data shown in SM3.

File Name: Peer Review File

Supplementary Information

Supplementary Figures

Supplementary Figure 1

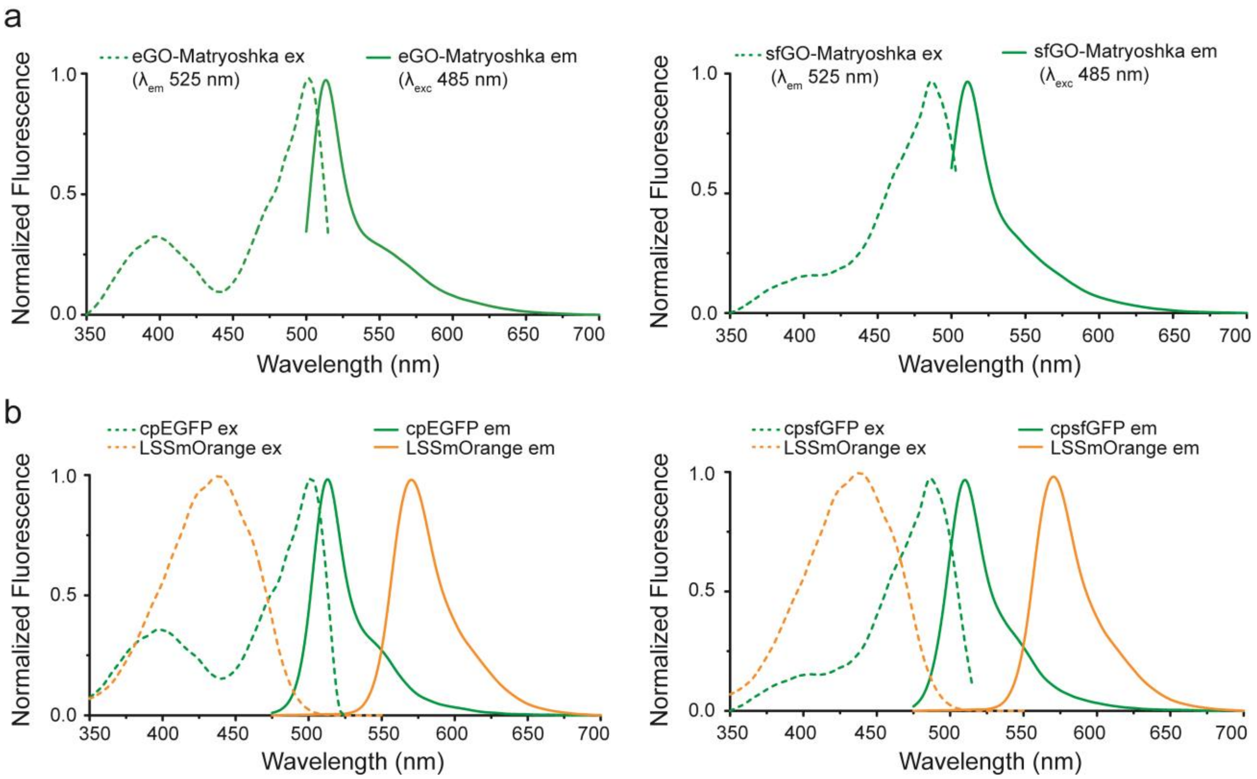

Steady-state fluorescence spectra of GO-Matryoshka variants in comparison to individual FPs. (a) Normalized steady-state fluorescence excitation (dashed line) and emission (solid line) of eGO-Matryoshka (left) and sfGO-Matryoshka (right). Green emission with minimal cross-excitation of LSSmOrange at λ_{exc} 485 nm was observed. (b) Individual FP normalized steady-state fluorescence excitation (λ_{em} 525/ 570 nm; dashed line) and emission (λ_{exc} 440 nm; solid line) of cpEGFP and LSSmOrange (left) and cpsfGFP and LSSmOrange (right). Note, all cpFPs and GO-Matryoshka versions shown here carry the LE/LP flanking amino acid combination. Saturating pH conditions were used for maximal FI. Data were normalized to the maximum FI.

Supplementary Figure 2

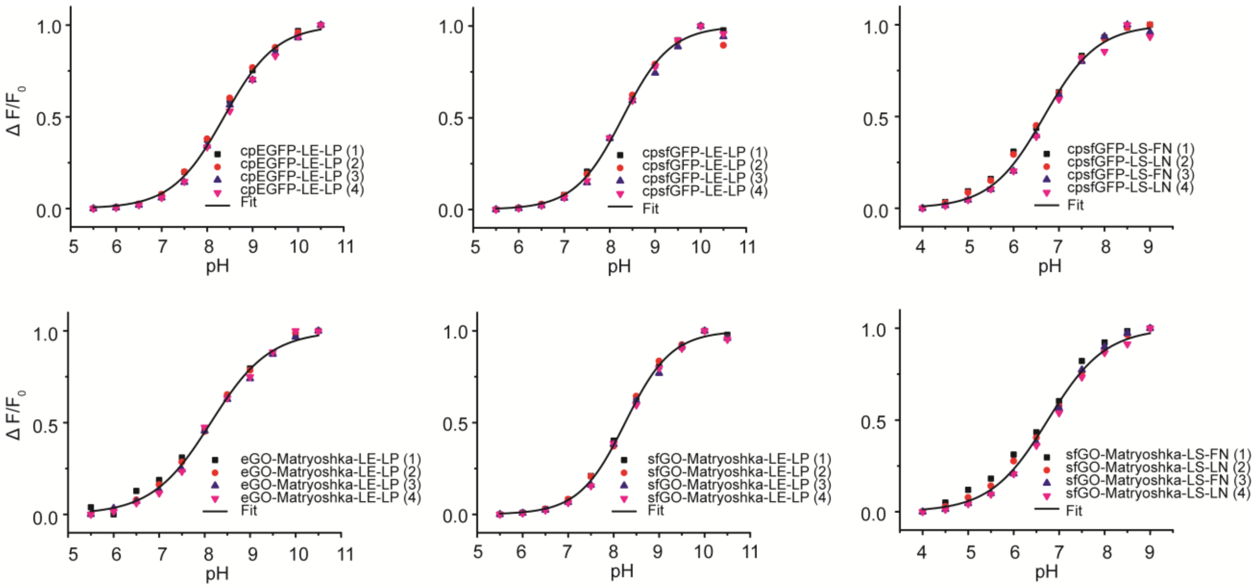

pH-titration curves of individual cpFP or GO-Matryoshka variants with different N- and C-terminal residue combinations. Individual curves represent independent biological replicates. pK_a values retrieved from the fits are listed in Supplementary Table 1.

Supplementary Figure 3

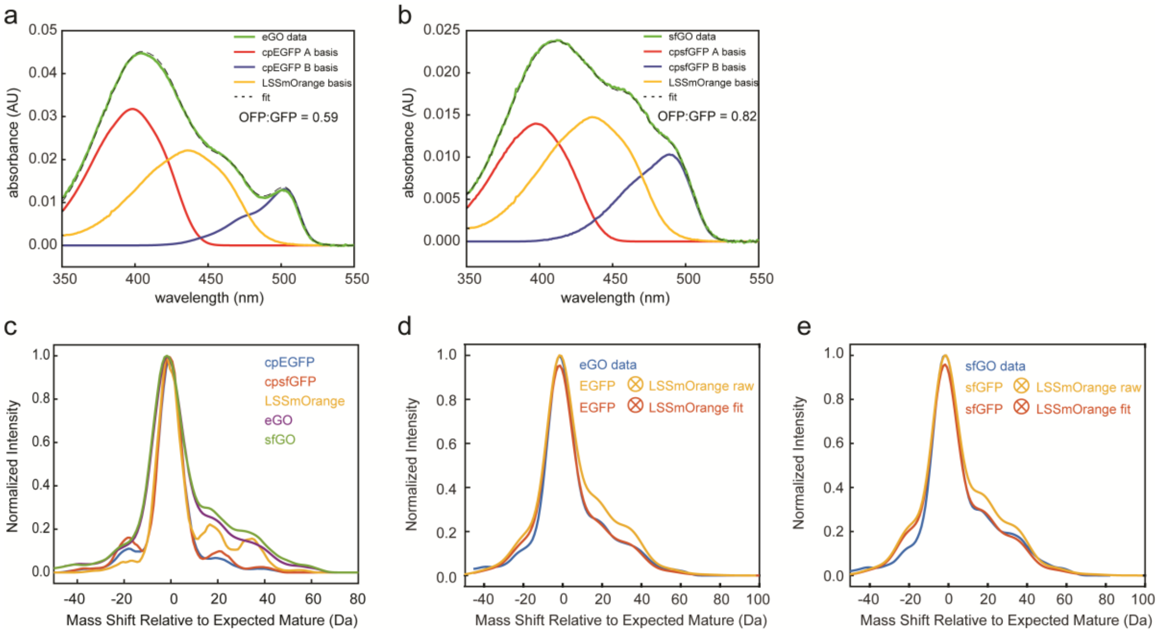

Maturation efficiency of cpFPs, LSSmOrange and GO-Matryoshka chromophores. (a, b) Visible absorbance spectra of eGO (a) and sfGO (b) were fitted with linear combinations of the constituent FP basis spectra. (c) Deconvoluted intact protein ESI mass spectra of single and nested FP species. The abscissa is scaled such that 0 represents the anticipated protein mass for full maturation of all chromophores and loss of the N-terminal methionine. The smaller peripheral peaks may result from immature chromophore species, salt adducts, methionine oxidation etc. (d, e) GO-Matryoshka mass spectra with the raw and fitted convolutions of the LSSmOrange mass spectrum numerically convolved with cpEGFP and cpsfGFP mass spectra, respectively.

Supplementary Figure 4

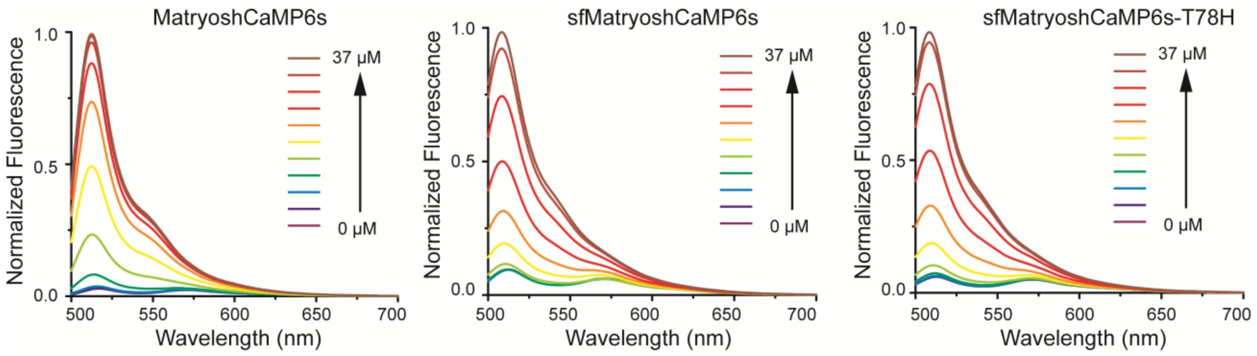

Steady-state fluorescence spectra (λ_{exc} 485 nm) of MatryoshCaMP6s, sfMatryoshCaMP6s and sfMatryoshCaMP6s-T78H calcium titration (0, 0.02, 0.03, 0.06, 0.09, 0.13, 0.20, 0.31, 0.52, 1.2, 37 µM).

Supplementary Figure 5

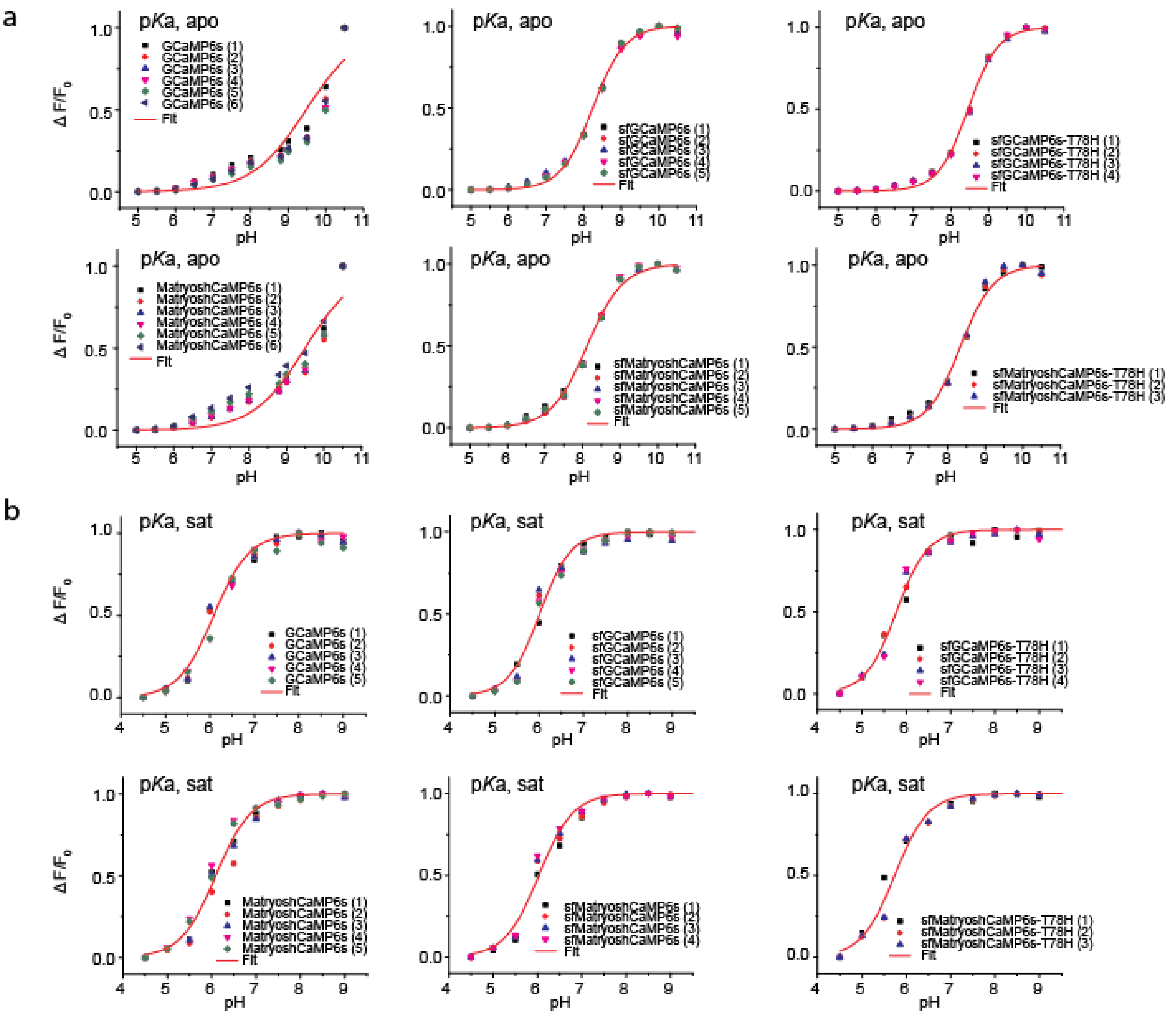

pH-titration curves of calcium sensor variants. Indicated GCaMP6s and MatryoshCaMP6s variants under calcium-free conditions ((a) p$K_{a,apo}$; 5 mM EGTA) and saturating calcium conditions ((b) p$K_{a,sat}$; 5 mM $CaCl_2$) calcium conditions (λ_{exc} 485 nm; λ_{em} 510 nm). Individual curves represent independent biological replicates. Note, GCaMP6s and MatryoshCaMP6s did not reach pH saturation under calcium-free conditions at maximal pH tested (pH 10.5). The resulting p*K*a values are listed in Table 1.

Supplementary Figure 6

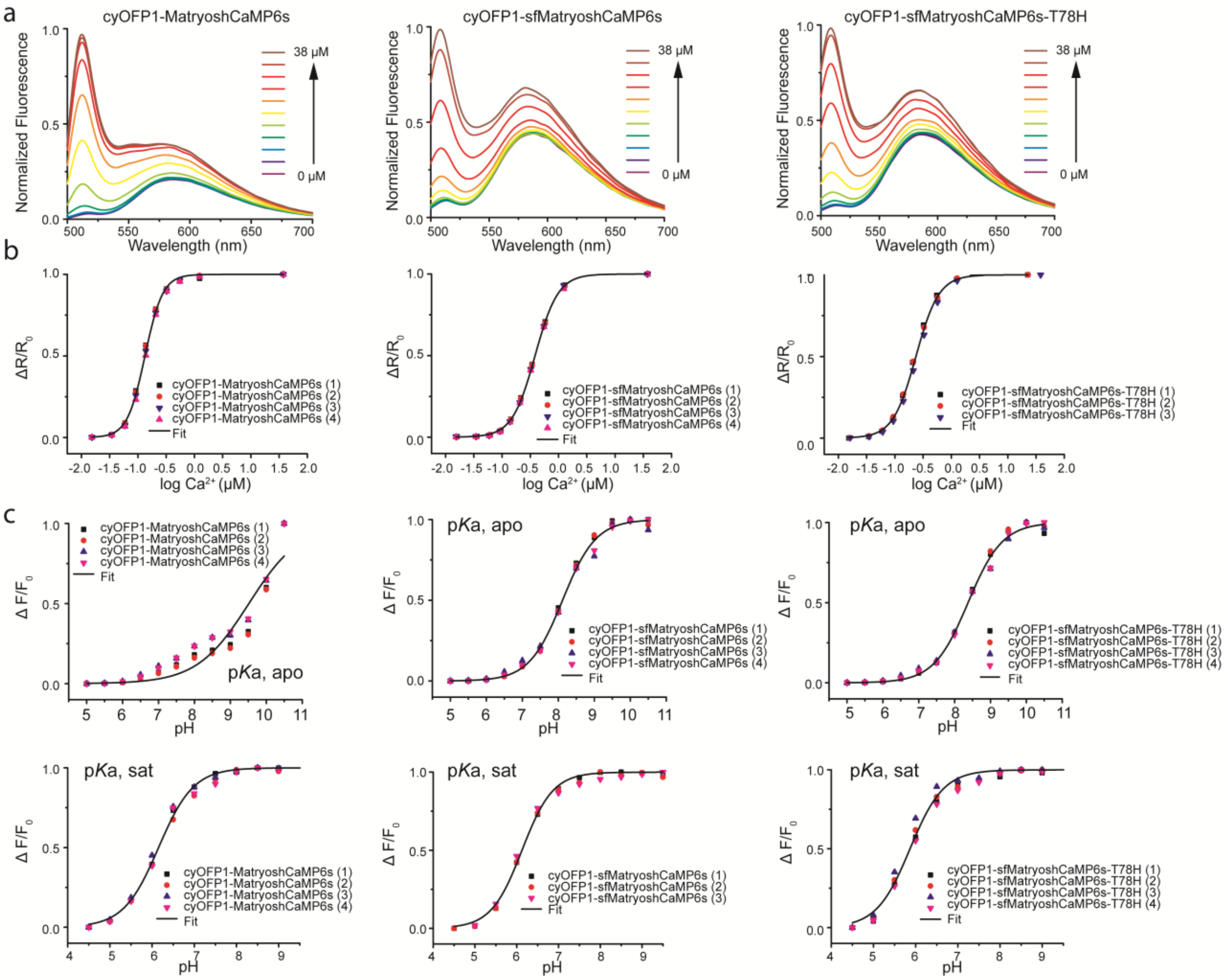

CyOFP1-MatryoshCaMP6s iteration analysis. The cyOFP1-MatryoshCaMP6s set includes variants of the MatryoshCaMP6s set, where LSSmOrange has been replaced by CyOFP1 (ref. [1]). (a) Steady-state fluorescence spectra (λ_{exc} 485 nm) of sensors titrated with free calcium (0, 0.02, 0.04, 0.06, 0.09, 0.14, 0.21, 0.32, 0.55, 1.2, 38 μM). Data were normalized to the maximum FI. (b) Calcium-affinity titrations ($R = FI_{510nm}/FI_{585nm}$) corresponding to the spectra in (b). (c) pH-titration curves under calcium-free conditions ($pK_{a,apo}$; 5 mM EGTA) and saturating calcium conditions ($pK_{a,sat}$; 5 mM $CaCl_2$) calcium conditions (λ_{exc} 485 nm; λ_{em} 505/510 nm). Individual curves represent independent biological replicates. The resulting p*K*a values are listed in Supplementary Table 2.

Supplementary Figure 7

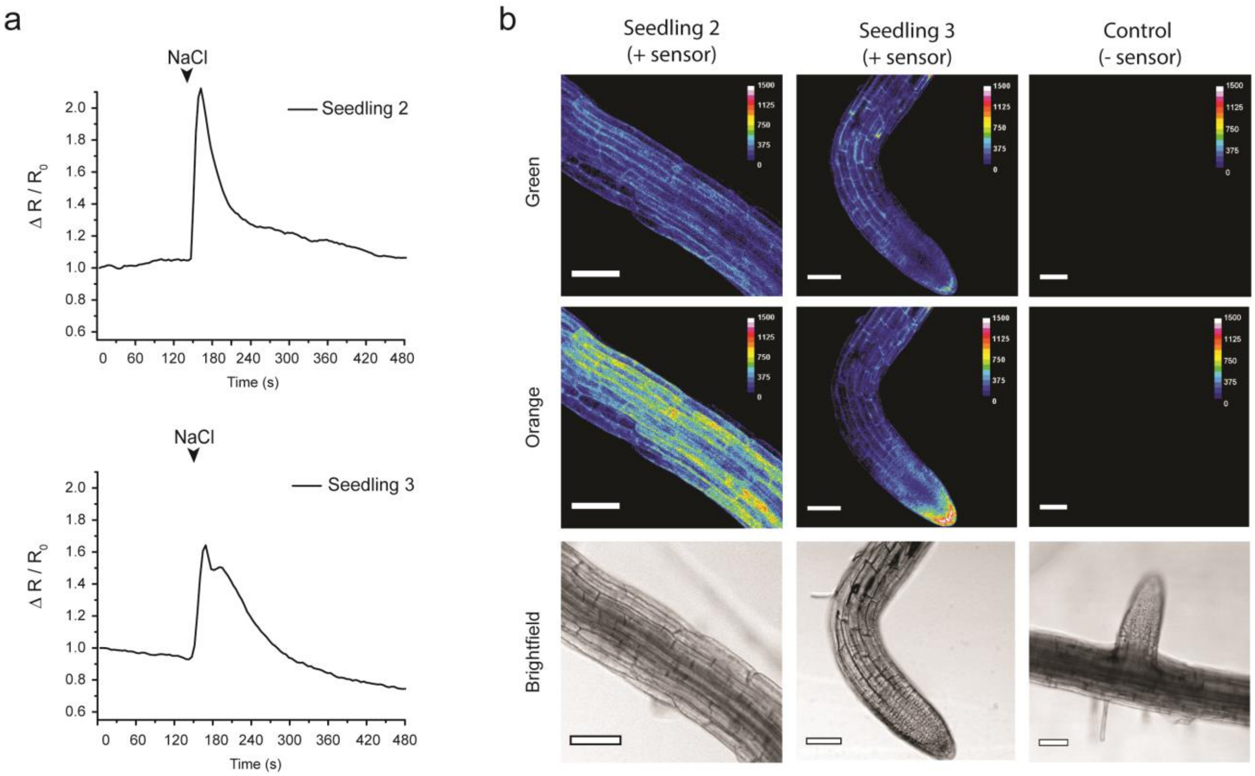

Arabidopsis seedlings expressing MatryoshCaMP6s consistently report root cytosolic calcium elevations in response to salt shock. (a) Graphs show normalized ratio of green to orange mean FI values across entire field of view. Arrowheads mark the time points (150 and 155 s, seedling 2 and seedling 3 respectively) that roots were exposed to salt shock by addition of NaCl to the growth media. Final NaCl concentrations in reservoirs were estimated to be between 10-25 mM. Samples were co-excited with 440 and 488 nm lasers. Emission intensities were simultaneously collected at 500-540 nm (green channel) and 570-650 nm (orange channel). (b) Average z-stack projections of confocal images showing *Arabidopsis* lateral roots. Seedling 2 and 3 refer to panel a) and are illustrated in comparison to a control seedling without sensor as autofluorescence control before NaCl treatment (t0). Note, there was no detectable autofluorescence under the imaging conditions tested. Seedlings were co-illuminated with 440 and 488 nm lasers. Emission intensities were simultaneously collected at 500-540 nm (Green Channel; top row) and 570-650 nm (Orange Channel; middle row) and are shown in 16-color lookup table with FI indicated in arbitrary units. Bright field images for each seedling are shown (single slice, bottom row). Seedlings were prepared and treated as in Fig. 3. Scale bar indicates 50 μm.

Supplementary Figure 8

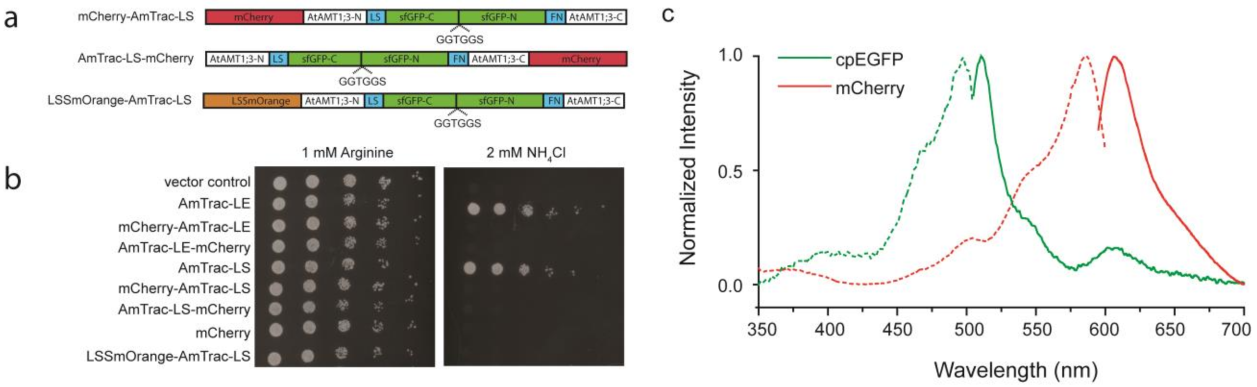

Terminal fusion of mCherry to AmTrac. (a) Schematic representation of AmTrac-LS with N-terminal and C-terminal mCherry fusion and N-terminal LSSmOrange fusion, respectively. (b) Complementation of the yeast *Δmep1,2,3* mutant transformed with indicated sensors grown on solid media with indicated N sources. Arginine served as growth control. The columns of plated cells represent 1:10 dilutions. (c) Spectral analysis of the cpEGFP (green) and mCherry (red) fluorescence of AmTrac-LS-mCherry. Excitation (dashed lines) was recorded at λ_{em} = 514 nm for green and at λ_{em} = 610 nm for red emission. Emission (solid line) was recorded at λ_{exc} = 485 nm for green and at λ_{exc} = 585 nm for red fluorophores, respectively. Values relate to the highest values.

Supplementary Figure 9

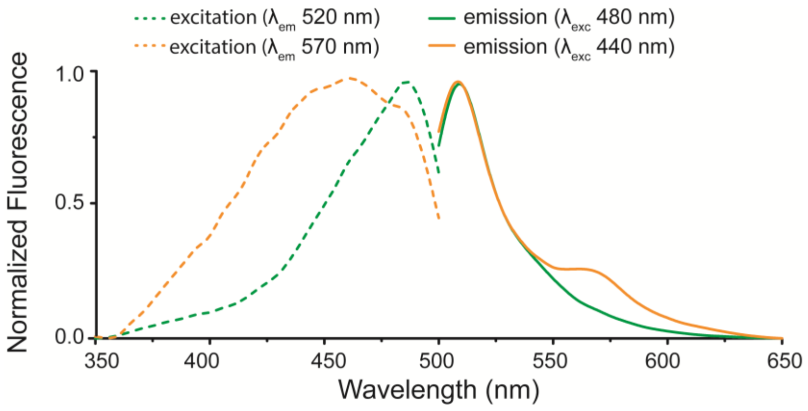

Normalized steady-state fluorescence excitation (λ_{em} 520/ 570 nm; dashed line) and emission (λ_{exc} 440/480 nm; solid line) of AmTryoshka1;3-GS. Data were normalized to the maximum FI.

Supplementary Figure 10

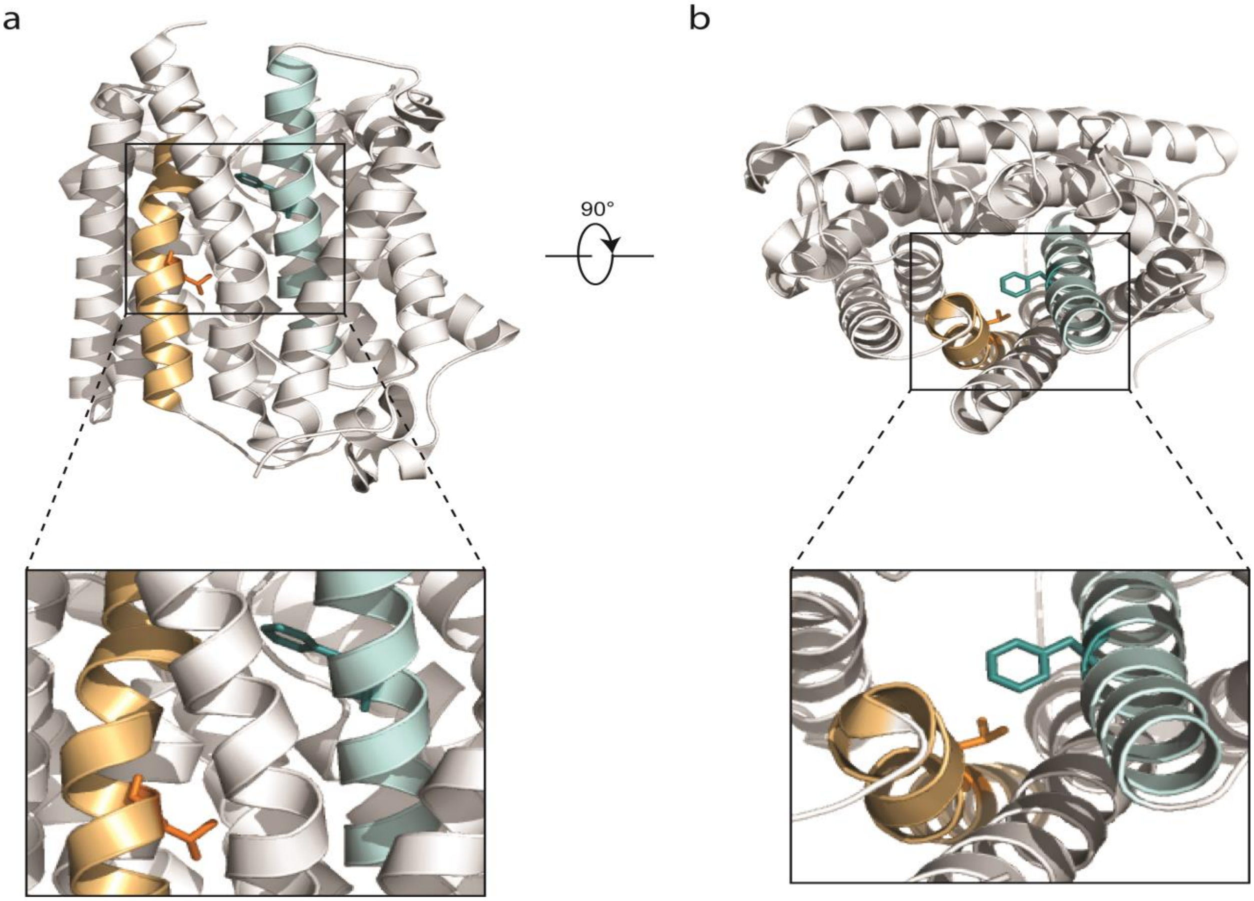

Chrystal structure of AfAMT1 with highlighted suppressor mutations. Cartoon illustration of F138 (cyan) and L255 (orange) position in side (a) and top view (b) of AMT monomer (AfAMT1 was used as proxy; PDB: 2B2F). Note, both residues point towards the ammonium transporter pore.

Supplementary Figure 11

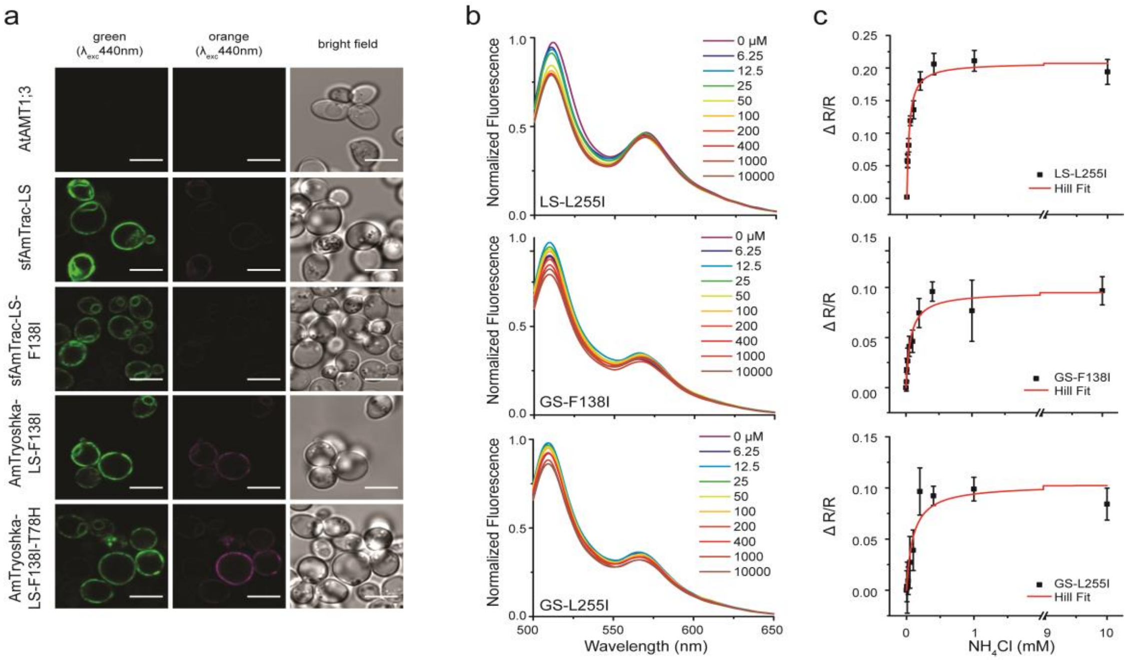

AmTryoshka1;3 set characterization. (a) Confocal z-sections of yeast *Δmep1,2,3* mutant transformed with AtAMT1;3 as autofluorescence control or indicated sensor variants to compare the effects of the F138I suppressor mutation and LSSmOrange insertion. Scale bar = 5µm. (b) Steady-state emission spectra of ammonium titration (0-10 mM) for AmTryoshka1;3-LS-L255I, -GS-F138I and -GS-L255I with λ_{exc} 440 nm (normalized to the maximum intensity). (c) Corresponding titration curve of the fluorescence response ΔR/R (R = FI_{510nm}/FI_{570nm}) (black square) and Hill fit (red line). Data were corrected for fluorescence bleed-through (bleed-through factor 0.08) and normalized to water-treated controls (mean ± s.e.m. *n* = 6 for LS-L255I, *n* = 4 for GS-F138I, *n* = 3 for GS-L255I). Affinities for ammonium transport derived from Hill plot are illustrated in Supplementary Table3.

Supplementary Figure 12

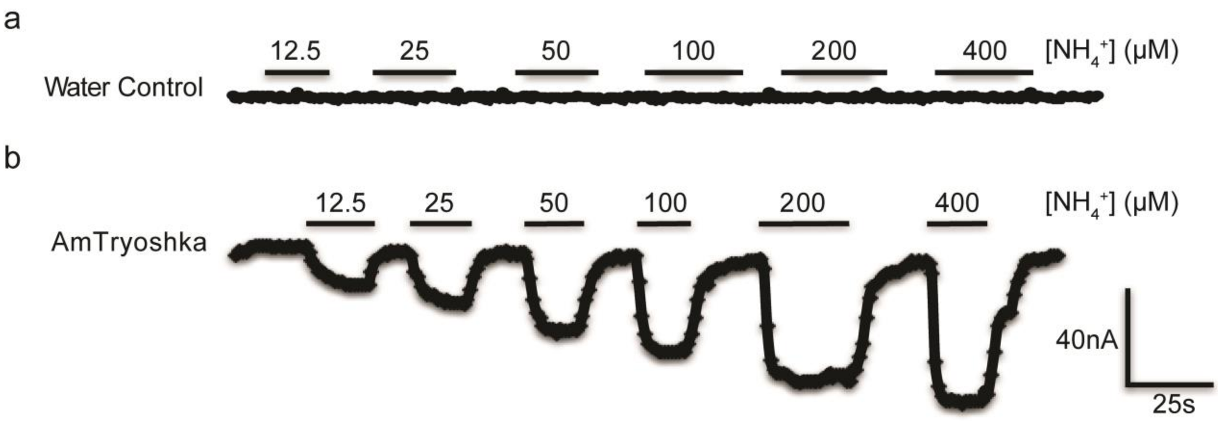

Concentration dependence of AmTryoshka1;3-LS-F138I-T78H-mediated inward currents induced by NH_4^+ in *Xenopus* oocytes using two-electrode-voltage-clamping. Currents recorded in single oocytes injected with water (control (a)) or AmTryoshka1;3-LS-F138I-T78H (b) during perfusion with NH_4^+ at the indicated concentrations. Oocytes were voltage clamped at -120 mV. The experiment was repeated in three individual oocytes.

Supplementary Tables

Supplementary Table 1 p*K*a values of individual cpFP or GO-Matryoshka variants with different N- and C-terminal residue combinations

	N-Terminus	C-Terminus	pK_a
cpEGFP	LE	LP	8.37 ± 0.02
eGO-Matryoshka	LE	LP	8.35 ± 0.01
cpsfGFP	LE	LP	8.28 ± 0.02
sfGO-Matryoshka	LE	LP	8.25 ± 0.01
cpsfGFP	LS	FN	6.67 ± 0.02
sfGO-Matryoshka	LS	FN	6.75 ± 0.02

p*K*a values retrieved from the pH titration fits (λ_{exc} 485 nm; λ_{em} 510/515 nm) in Supplementary Figure 2 (mean ± s.e.m; *n* = 4).

Supplementary Table 2 cyOFP1-based MatryoshCaMP6s properties

Sensor	K_d (nM) $\Delta F/F_0$	K_d (nM) $\Delta R/R_0$	p$K_{a,\ apo}$	p$K_{a,\ sat}$	Dynamic range $_{485\ exc}$ $\Delta R/R_0$	Dynamic range $_{485\ exc}$ $\Delta F/F_0$
cyOFP1-MatryoshCaMP6s	133±3	180±7	~ 8 *	6.15±0.02	20.92±0.79	44.85±2.03
cyOFP1-sfMatryoshCaMP6s	384±2	476±3	8.12±0.01	6.13±0.02	6.50±0.10	10.83±0.22
cyOFP1-sfMatryoshCaMP6s-T78H	236±10	290±9	8.38±0.01	5.87±0.02	10.79±0.63	17.64±0.99

Calcium affinities and p*K*a values retrieved from the calcium and pH titration fits (λ_{exc} 485 nm; λ_{em} 510/515 nm) in Supplementary Figure 6 (mean ± s.e.m; *n* = 3- 4). * Value is estimate, since saturation was not reached at the maximal pH tested here (pH 10.5)

Supplementary Table 3 Affinities for ammonium transport of AmTryoshka1;3 set

Sensor	$K_{0.5}$ (μM)
AmTryoshka-LS-F138I	84.6 ± 20.4
AmTryoshka-LS-F138I-T78H	62.4 ± 8.6
AmTryoshka-LS-255I	39.6 ± 9.4
AmTryoshka-GS-F138I	68.9 ± 26.7
AmTryoshka-GS-L255I	98.8 ± 45.7
AmTrac	55 ± 7*

Affinities for ammonium transport derived from Hill plot of ammonium titration in Figure 5e and Supplementary Figure 11c (* from[2])

Supplementary Table 4 List of primers

Name	Sequence
AmLS_sfGFPcp_FW	GTC CTC GTC GTG GTC GGT TCG AGA AAT TGT CCA ACG TGT ATA TTA CCG CGG
AmGS_sfGFPcp_FW	GTC CTC GTC GTG GTC GGT TCG AGA AAG GTA GTA ACG TGT ATA TTA CCG CGG
AmFN_sfGFPcp_RV	GCG CAG AGC AAT AGC GCG ACC ACC ATT AAA GTT ATA TTC CAG TTT ATG CCC
sfLS-LSSmO_FW	GTC GTG GTC GGT TCG AGA AAT TGT CCA ACG TGT ATA TTA CCG CGG
sfLS-LSSmO_RV	CCG CGG TAA TAT ACA CGT TGG ACA ATT TCT CGA ACC GAC CAC GAC
sfAmTrac-F138I_FW	CTT CCT CTA CCA ATG GGC GAT CGC AAT CGC GGC CGC TGG
sfAmTrac-F138I_RV	CCA GCG GCC GCG ATT GCG ATC GCC CAT TGG TAG AGG AAG
sfAmTrac-L255I_FW	GTC TTA GGA ACC TTC CTC ATA TGG TTT GGA TGG
sfAmTrac-L255I_RV	CCA TCC AAA CCA TAT GAG GAA GGT TCC TAA GAC
LS-cpsfGFP_FW	GGC ATC ATC ATC ATC ATC ATA GCA GCG GCT TGT CCA ACG TGT ATA TTA CCG CGG
LS-cpsfGFP_RV	CCG CGG TAA TAT ACA CGT TGG ACA AGC CGC TGC TAT GAT GAT GAT GAT GAT GCC
cpsfGFP-FN_FW	GGG CAT AAA CTG GAA TAT AAC TTT AAT TAA CTC GAG GAT CCG GCT GC
cpsfGFP-FN_RV	GCA GCC GGA TCC TCG AGT TAA TTA AAG TTA TAT TCC AGT TTA TGC CC
LSSmOr-pET15b_InF_1st_FW	GGC ATC ATC ATC ATC ATC ATA GCA GCG GCA TGG TGA GCA AGG GCG AGG A
LSSmOr-pET15b_InF_1st_RV	TTA CTT GTA CAG CTC GTC CAT GCC G
LSSmOr-pET15b_InF_2nd_FW	agg aga tat aCC ATG GGG CAT CAT CAT CAT CAT CAT AGC AGC
LSSmOr-pET15b_InF_RV	CAG CCG GAT CCT CGA GTT ACT TGT ACA GCT CGT CCA TGC CG
GCaMP6s-EGFPcp-LSSmO-InF_FW	GTACAAGGGCGGTACCATGGTGAGCAAGGGCGAGGA
GCaMP6s-EGFPcp-LSSmO-InF_RV	CACCATGCTCCCTCCCTTGTACAGCTCGTCCATGCC
sfGFPcp-XhoI-M13-InF_FW	CTGAGCTCACTCGAGAACGTGTATATTACCGCGGAT
sfGFPcp-LP-CaM_RV	TCAGTCAGTTGGTCCGGCAGGTTATATTCCAGTTTATGCCCC

CaM-LP-sfGFPcp_FW	GGCATAAACTGGAATATAACCTGCCGGACCAACTGACTGA
CaM-pRSET-HindIII-InF_RV	CAGCCGGATCAAGCTTCGAATTGC
pET-EGFPcp-new_InF_FW	TAGCAGCGGCCTCGAGAACGTCTATATCAAGGCCGAC
pET-EGFPcp-new_InF_RV	CAGCCGGATCCTCGAGTTACGGCAGGTTGTACTCCAGCTTGT
LE-cpsfGFP_FW	GGC ATC ATC ATC ATC ATC ATA GCA GCG GCC TCG AGA ACG TGT ATA TTA CCG CGG
LE-cpsfGFP_RV	CCG CGG TAA TAT ACA CGT TCT CGA GGC CGC TGC TAT GAT GAT GAT GAT GAT GCC
cpsfGFP-LP_FW	GGG CAT AAA CTG GAA TAT AAC CTG CCG TAA CTC GAG GAT CCG GCT GC
cpsfGFP-LP_RV	GCA GCC GGA TCC TCG AGT TAC GGC AGG TTA TAT TCC AGT TTA TGC CC
cpGFP-IF1	GGAGGGAGCATGGTGAGC
cpGFP-IR1	GGTACCGCCCTTGTACAG
sfcpGFP-IF1	GGCGGCAGCGCGAGCCAG
sfcpGFP-IR1	GGTGCCGCCATACAGTTC
cyOFP1-F1h1	CTGTACAAGGGCGGTACCATGGTGAGCAAGGGCGAG
cyOFP1-F1h2	GAACTGTATGGCGGCACCATGGTGAGCAAGGGCGAG
cyOFP1-R1h1	GCTCACCATGCTCCCTCCCTTGTACAGCTCGTCCATGCC
cyOFP1-R1h2	CTGGCTCGCGCTGCCGCCCTTGTACAGCTCGTCCATGCC

Supplementary Note 1

Determination of chromophore maturation efficiency

In order to assess the effects of the Matryoshka concept on the maturation efficiencies of the constituent fluorescent proteins (FPs) we took a two-pronged approach by analyzing the separate single FP species (cpEGFP, sfGFP, and LSSmOrange) and the derivative Matryoshkas (ie. eGO and sfGO) by visible absorbance spectroscopy and intact protein mass spectrometry. All analyzed proteins were purified first by Ni^{+2}-affinity chromatography and then by anion exchange chromatography.

GFP is well known to exhibit two pH-sensitive absorbance bands which correspond to the protonated neutral (A-state) and deprotonated ionized (B-state) forms of the chromophore. We collected absorbance spectra at a range of pH values to vary the relative magnitude of these two bands and extrapolated to the intrinsic basis spectra for each form for both cpEGFP and cpsfGFP. LSSmOrange has only one peak which is largely pH insensitive and was used directly as the LSSmOrange basis spectrum. The weighting of extinction coefficients was performed using the standard base denaturation method introduced by Ward et al[3].

With the basis spectra in hand we measured the absorbance spectra of the eGO and sfGO Matryoshka variants and performed least squares fitting using Matlab to determine the relative concentrations of mature GFP and OFP (orange FP, here LSSmOrange) chromophores (Supplementary Fig 3a, b). Our findings suggest that the ratios of OFP:GFP are not exactly 1:1 as would be expected for complete maturation of both species. Instead, we observe that there is somewhat less mature LSSmOrange than GFP. This fact, on its own, does not limit the usefulness of the internal reference provided that the fraction of immature LSSmOrange is consistent and ideally reflects the maturation efficiency of solitary LSSmOrange itself.

To obtain a more complete picture including both mature and immature species we turned to intact protein mass spectrometry (MS). The chromophore maturation process involves a dehydration and oxidation step, which collectively results in a loss of 20 Da. This mass shift can be resolved with high resolution intact protein ESI-MS. Purified cpEGFP, cpsfGFP, LSSmOrange, eGO, and sfGO were all analyzed by LCMS with a Bruker micrOTOF-Q II mass spectrometer. The resulting mass spectra were processed by MaxEnt for deconvolution of the multiple charge states. All samples resulted in a major mass peak corresponding to N-terminal methionine loss and the expected mass shift due to chromophore maturation (-20 Da for single

FP species, -40 Da for nested FP species) (Supplementary Fig 3c). Additionally, there were a number of smaller peripheral peaks, which may have arisen from immature chromophore species, salt adducts, methionine oxidation, etc. Those proteins containing LSSmOrange (LSSmOrange, eGO, and sfGO) in particular showed a greater abundance of these side peaks, possibly owing to incomplete chromophore maturation.

The central question is whether the Matryoshka strategy (ie. nesting an LSSmOrange inside a single FP biosensor) compromises the chromophore maturation efficiency. The absorbance analysis and the presence of the major peak at the expected masses of the GO species (Supplementary Fig 3c) suggest that a significant portion of LSSmOrange is mature. However, for a more quantitative perspective we took a data-driven approach to the mass spectrometry data.

If the FPs do not influence the maturation properties of each other then one expects that the mass spectrum of the nested FP species should be the convolution of the two single spectra. For example, eGO should be the spectrum of cpEGFP convolved with the spectrum of LSSmOrange. The corresponding spectra were numerically convolved in Matlab and are plotted against the spectra of eGO and sfGO (Supplementary Fig 3d, e; compare yellow traces to blue traces). There is clearly close correspondence in the spectral shape.

We took this analysis one step further by decomposing the LSSmOrange spectrum (Supplementary Fig 3c) into the projected correct peak and the remaining peripheral peaks. The "correct" spectral peak is centered at the expected mature protein mass and has a width dictated by counting statistics and the natural isotope abundances. The "peripheral" spectrum was simply the LSSmOrange spectrum minus the "correct" spectrum. Then the numerical convolution was calculated for the GFP spectrum and a linear combination of the "correct" and "peripheral" LSSmOrange spectra. The best fits were determined by least-squares optimization in Matlab and are shown as the red traces (Supplementary Fig 3d, e). This fitting procedure results in nearly perfect reproduction of the GO spectra. In both cases the best fit actually has a slight enrichment of the "correct" peak relative to the original LSSmOrange spectrum.

Overall these results indicate that the Matryoshka construct leads to no decrease in the extent of LSSmOrange maturation and that the OFP:GFP disparity found from the absorbance measurements is likely a consequence of incomplete maturation which is probably also present in solitary LSSmOrange.

Supplementary Note 2

Predicted determinants of dynamic range

We used the data of the calcium sensors GCaMP6s and sfGCaMP6s to investigate determinants of the dynamic range, with the intention of developing a more generalizable approach to sensor engineering using a mathematical model. With a set of absorbance and fluorescence excitation measurements performed at a range of pH values under calcium-free and calcium-saturated conditions, we have been able to examine in detail the factors influencing the biosensor dynamic ranges. Previous efforts to understand the origins of the sensor response have focused primarily on changes in fluorescence quantum yields and differential pKa's between the apo- and ligand-bound protein[4–6]. Our analysis has revealed that, in addition to these two identified mechanisms, a third factor - internal acid-base equilibrium - is of considerable importance to the dynamic range.

Fluorescent proteins (FPs) are routinely described to have a pK_a that characterizes the transition between the neutral (often dark) and anionic (usually emissive) forms of the chromophore as a function of pH. A simple single-site titration is implicit in this treatment, yet, the coupling between the ionization state of the chromophore and the surrounding ionizable amino acid side chains can be quite significant. Consequently, these titrations can exhibit complex behavior, including negative cooperativity, response plateau regions, or apparent mixed states in limits of high or low pH. The original wild-type *Aequoria victoria av*GFP, in fact, dramatically exemplifies strong coupling to E222, resulting in a nearly flat pH response from pH 7-11. Our analysis indicates that site coupling plays a prominent role in these calcium biosensors as a major determinant of sensor performance.

We constructed a simple model to better understand the relationship among the three central mechanisms of sensor dynamic range: 1) the difference between the pK_a's (or apparent pK_a's, as shall be further clarified) between the apo and saturated species, 2) the difference in quantum yield of the emissive state between the apo and saturated species, and 3) the internal ionization equilibrium of the apo species. A two-site model (Supplementary Figure 13a) captures the essence of the internal buffering process and has been shown in previous studies to satisfactorily explain many of the anomalous pH titration behaviors in fluorescent proteins[7–9]. This two-site model postulates a secondary ionizable site, “X”, whose ionization state is energetically coupled to the chromophore ionization. That is, the propensity of the chromophore to become

deprotonated is different depending on whether "X" is ionized or neutral. When the magnitude of this coupling is large, one observes plateaus in the pH titrations as exemplified in Supplementary Figure 13b. In this pH range (pH ~8-9) the behavior is dominated by the internal buffering process, independent of the external pH. The role of "X" is clearly filled by E222 in *av*GFP. Its identity in single-FP sensors is less clear, but is likely played by nearby ionizable residues and perhaps accounts, in part, for the performance sensitivity to the flanking amino acid sequences.

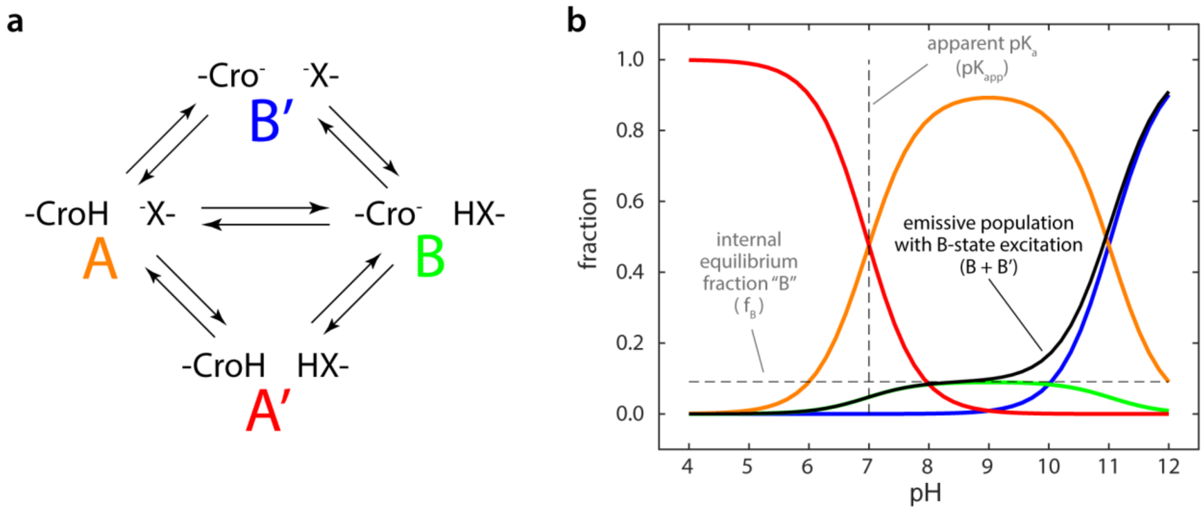

Supplementary Figure 13. Model of chromophore coupling and the role of acid-base equilibrium for sensor performance. (a) The scheme illustrates the model of coupling between the chromophore, 'Cro', and a secondary ionizable site, 'X'. The fully protonated species A' has two pH dependent deprotonation pathways, with acid dissociation constants $K_{A'A}$ and $K_{A'B}$ for A'→A and A'→B, respectively. The equilibrium between A and B is pH independent, since the proton transfer is internal. Strong inter-site coupling pushes the second deprotonation event (transitions to B') to higher pH, thus creating a plateaued region, for example, between pH 8 and 9. For more detail see Oltrogge et al[9]. (b) The equilibrium between A and B is obtained directly from thermodynamic loop closure, therefore the fraction of the emissive B state, f_B, can be straightforwardly calculated as $f_B = \frac{K_{A'B}}{K_{A'B} + K_{A'A}}$. The apparent p$K_a$ of the transition to this plateaued region can be defined as the pH at which B is at half the plateau level and is given by, $pK_{app} = -\log_{10}(K_{A'B} + K_{A'A})$. The titration panel shows the calculated fraction of each species as a function of pH. The calculation was performed with p$K_{A'B}$ = 8.0, p$K_{A'A}$ = 7.0, and a site-coupling energy of 3*ln(10) RT.

The data for all the apo calcium biosensors revealed a plateau at high pH in the population of deprotonated chromophore short of 100%. This clearly indicates that the chromophores are experiencing internal buffering, because the chromophore ionization would otherwise proceed to completion. Importantly this sub-saturating limit reduces the possible amount of the emissive "B" state (f_B), thus limiting the fluorescence and thereby boosting the dynamic range by a factor of $1/f_B$ for any pH at or below the plateau region. The relative quantum yield (Φ_{apo}/Φ_{sat}) serves a similar role, by increasing the dynamic range in a pH-independent manner.

The calcium-saturated species closely conform to a simple single-site titration (*i.e.* they reach complete deprotonation in a sigmoidal fashion). The apo species do not. However, with their sizable site couplings, the first transition is indeed well described in terms of a single-site titration to the sub-saturating limit, f_B. Furthermore, for the sake of simplicity we are assuming excitation of only the deprotonated GFP absorbance band and that the intrinsic extinction coefficients of the apo and ligand-bound deprotonated species are equal. Using these assumptions, we can derive an expression for the dynamic range as a function of pH,

(Eq. 1)

$$\left(\frac{\Delta F}{F_0}\right) = \left(\frac{F_{sat} - F_{apo}}{F_{apo}}\right) = \frac{f_{SS}(pH, pK_{a,sat}) - f_{SS}(pH, pK_{app,apo}) * f_B * \left(\frac{\Phi_{apo}}{\Phi_{sat}}\right)}{f_{SS}(pH, pK_{app,apo}) * f_B * \left(\frac{\Phi_{apo}}{\Phi_{sat}}\right) + N_0}$$

where $pK_{a,\,sat}$ is the pK_a of the ligand saturated species, $pK_{app,\,apo}$ is the apparent pK_a of the apo species (explicitly defined in Supplementary Figure 13b), f_{SS} is a function containing the pH dependence of a single-site deprotonation as $f_{SS}(pH, pK_a) = \frac{1}{1 + 10^{(pK_a - pH)}}$, f_B is the internal equilibrium factor in the apo species (explicitly defined in Supplementary Figure 13b), Φ_{apo}/Φ_{sat} is the relative quantum yields of the emissive states of the apo and saturated species, and N_0 is the relative level of background noise.

For clarity of presentation Eq. 1 assumes excitation only of the deprotonated GFP absorbance band (B-state) and that the intrinsic B-state extinction coefficients for the apo and ligand-bound species are equal. Indeed, excitation only on the B-state is typical experimental practice with GFP-based biosensors. Extending the analysis to arbitrary wavelength requires that one take into account excitations of the protonated GFP absorbance band (A-state). The excited A-state is well-known to undergo an ultrafast excited state proton transfer (ESPT) process which culminates in B-state-like green fluorescence emission[10]. With complete knowledge of the basis spectra for all the optical species (ie. apo A-state, sat. A-state, apo B-state, and sat. B-state) and the fluorescence and ESPT quantum yields it is possible to derive a more comprehensive expression which maintains generality for arbitrary excitation wavelength (λ):

(Eq. 2)

$$F_{sat}(\lambda, pH) = f_{SS}(pH, pK_{a,sat}) * \varepsilon_{B,sat}(\lambda) * \phi_{B,sat} + \\ [1 - f_{SS}(pH, pK_{a,sat})] * \varepsilon_{A,sat}(\lambda) * \phi_{ESPT,sat} * \phi_{I^*,sat} + N_0$$

(Eq. 3)

$$F_{apo}(\lambda, pH) = f_{SS}(pH, pK_{a,apo}) * \varepsilon_{B,apo}(\lambda) * \phi_{B,apo} * f_B + \\ [1 - f_{SS}(pH, pK_{a,apo})] * \varepsilon_{A,apo}(\lambda) * \phi_{ESPT,apo} * \phi_{I^*,apo} + N_0$$

(Eq. 4)

$$\frac{\Delta F}{F_0} = \frac{F_{sat}(\lambda, pH) - F_{apo}(\lambda, pH)}{F_{apo}(\lambda, pH) + N_0}$$

where f_{ss} is the standard single site titration sigmoid for the deprotonated species, ε(λ) is the intrinsic extinction coefficient weighted basis spectrum for the corresponding species (e.g. apo A-state), and ϕ is the quantum yield either for fluorescence or excited-state proton transfer.

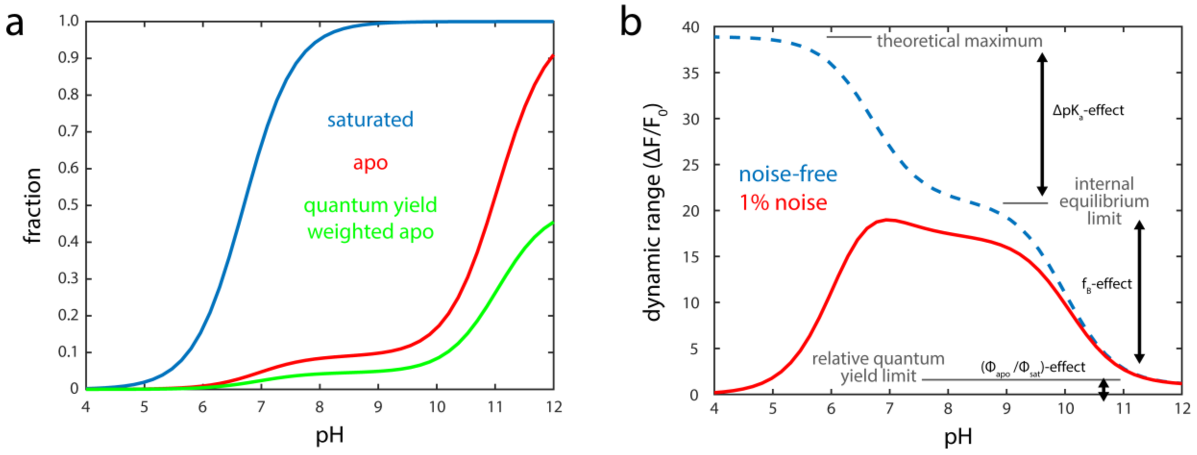

Supplementary Figure 14. Example titrations of pH and dynamic range. (a) Calculated example titrations for the ligand-saturated biosensor (blue, with p$K_{a,\, sat}$ = 6.7), the apo sensor (red, B + B' from Supplementary Figure 13), and the apo species weighted by the relative quantum yield (green, with Φ_{apo}/Φ_{sat} = 0.5). (b) Dynamic ranges derived from the example titrations calculated with saturated and quantum yield-weighted apo curves. It is important to note that the dynamic range effects are multiplicative, not additive, and thus the relative significance of each may not be visually apparent. Note also that for illustrative purposes the dynamic range curves are calculated explicitly from the model in Supplementary Figure 13 in order to include the eventual transition to B'. Implicit in Eq. 1 is the assumption that this final transition falls outside the pH region of practical interest (i.e., Eq. 1 is valid from pH 4- 9 in this example). The dashed blue line represents the noise free idealization and predicts that the dynamic range should improve asymptotically with decreasing pH. In actuality, however, the fluorescence intensity quickly approaches zero and will eventually be overwhelmed by background noise (red trace with 1% noise). This trade-off between increasing intrinsic dynamic range and decreasing signal-to-noise leads to a peaked value of realized dynamic range as a function of pH.

The theoretical maximum dynamic range can be obtained from Eq. 1 in the limit of acidic pH and zero noise.

(Eq. 5)

$$\left(\frac{\Delta F}{F_0}\right)_{\max} = \frac{10^{\Delta pK_a}}{\left(\Phi_{apo}/\Phi_{sat}\right) f_B} - 1$$

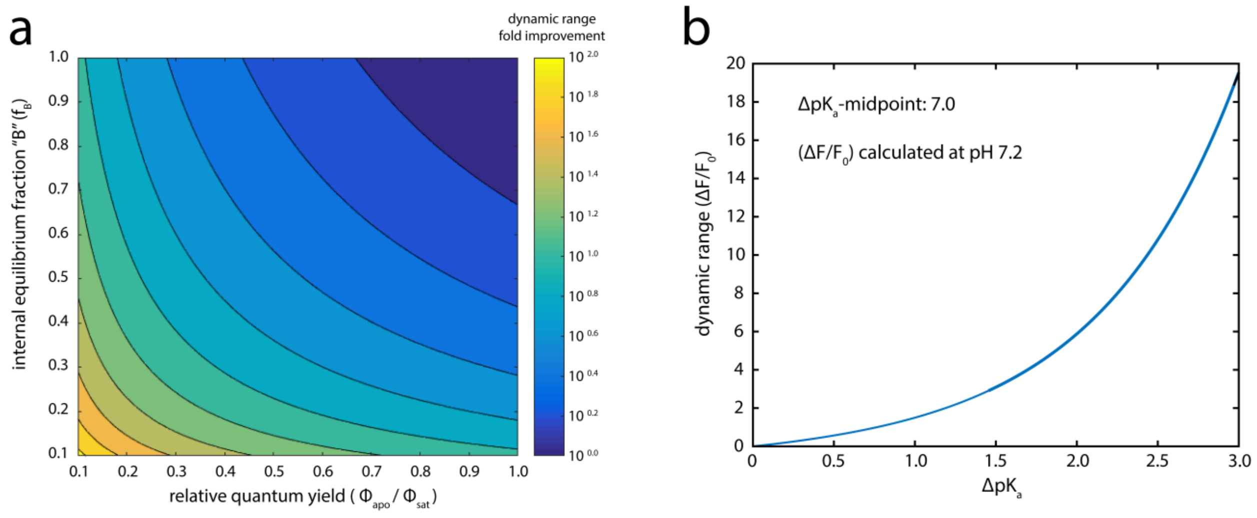

Supplementary Figure 15. Effect of the parameters f_B, (Φ_{apo}/Φ_{sat}), and ΔpK_a on the dynamic range. (a) Dependence of the dynamic range on f_B and (Φ_{apo}/Φ_{sat}). These parameters have a pH-independent effect, resulting in this being a general description. (b) Illustration of the functional form of the dynamic range due to ΔpK_a with a specific example because of the dependence on the ΔpK_a -midpoint and pH of observation.

To explore the practical implications of the above formulation, we tabulated the factors influencing the dynamic range for GCaMP6s and sfGCaMP6s (Supplementary Table 5). These two specific examples are broadly representative of the two categories of calcium sensors we built based on the original cpEGFP or cpsfGFP scaffolds, respectively.

Supplementary Table 5 Influencing Factors of the dynamic range (D.R.)

	GCaMP6s	sfGCaMPs
pK_a effects		
$pK_{a, sat}$	6.1	6.0
$pK_{app, apo}$	7.7	8.3
ΔpK_a	1.6	2.3
D.R. max. factor	39.8	199.5
D.R. factor at pH 7.2	2.9	11.8
quantum yield effects		
Φ_{apo}/Φ_{sat}	0.34	1.02
D.R. factor	3.0	1.0
internal equilibrium effects		
f_B	0.16	0.59
D.R. factor	6.3	1.7
relative contribution of D.R. factors at pH 7.2		
pK_a	20%	94%
Φ_{apo}/Φ_{sat}	22%	0%
f_B	58%	6%

There are a number of interesting implications, which follow from the itemized accounting of dynamic range. Based on the analyses, a key factor is the previously unappreciated internal equilibrium factor described here. GCaMP6s derives its large dynamic range by utilizing all three mechanisms: differential pK_a's and quantum yields of the ligand-saturated and apo species, and internal acid-base equilibrium. By contrast, sfGCaMP6s relies almost exclusively on the

differential pK_a's between the apo and saturated species. Due to the multiplicative nature of the factors, sfGCaMP6s could significantly gain dynamic range by even a small improvement of the relative quantum yield and internal equilibrium factors. The maximum pK_a-associated factors stand out for their extremely high values (Supplementary Table 5). This metric is rather deceptive, however, because it occurs in a limit of vanishing fluorescence intensity. In practice, when optimizing a biosensor, the realizable dynamic range also depends on the brightness and may be better served by improving other factors, i.e., relative quantum yield and internal equilibrium, which do not negatively impact brightness of the saturated species.

An important limitation of single-FP biosensors is their high sensitivity to the environmental pH. That is, the true signal due to the intended analyte can be confounded by fluctuations in the cellular pH. The severity of this problem is tied directly to the relative contribution of the three dynamic range determinants described herein. In particular, the larger the dynamic range contribution due to the differential pK_a the greater the sensitivity to pH will be. In contrast, the relative quantum yield and the internal equilibrium should not be significantly affected by pH changes. Consequently it may be an advantageous trade-off to have a biosensor that relies chiefly upon the latter two factors even at the cost of a lower overall dynamic range because its signal response would be largely immune to pH variation.

The model operates at a level of abstraction beyond the molecular specifics and does not pinpoint specific residues as targets for mutagenesis. Any given residue could in principle affect all three factors. Generically speaking, any residue with a plausible hydrogen bonding network extending to the phenolate of the chromophore is very likely to modify the internal acid-base equilibrium. The flanking amino acid residues are probably capable of filling this role. Other residues which are likely significant would be (by wtGFP number conventions) 222, 205, 203, and 65, as these are important mediators of internal hydrogen bond networks around the chromophore. It is possible that one could accentuate the sensors by breaking the internal H-bond network, which could favor a new network to either flanking amino acid or sensing domain residues. Further analyses, such as crystal structures of the MatryoshCaMP and AmTryoshka versions, as well as systematic site directed mutagenesis of the residues listed above using the methods described here may be a path to optimizing the sensors and to better understanding the molecular basis of the interplay of the three principal components that affect the dynamic range.

In summary, we identified the internal acid-base equilibrium as a major determinant of the dynamic range achievable by single-FP and, by extension, Matryoshka biosensors. Furthermore, this new factor has been placed in quantitative context with the previously known mechanisms of differential pK_a's and quantum yield differences between the ligand-saturated and apo species. The importance of internal acid-base equilibrium is underlined by its responsibility for the majority of the dynamic range of GCaMP6s, one of the most responsive biosensors available to date. This more refined understanding of dynamic range may help facilitate future sensor design and optimization.

Supplementary Note 3

Generation of AmTrac sensors based on cpsfGFP

For comparison of the AmTryoshka sensor series with their appropriate parent sensors and to assess the effects of cpsfGFP versus cpEGFP in the original AmTrac[2], variants that carried only cpsfGFP without the LSSmOrange (termed sfAmTrac) were generated. In AmTrac, we found that the amino acids flanking the cpsfGFP and connecting it to the AtAMT1;3 domain affected the sensors properties[2]. Thus, we maintained the flanking residue combination in the new sfAmTrac sensors: (1) sfAmTrac-LE (leucine/glutamate), (2) sfAmTrac-LS (leucine/serine) and (3) sfAmTrac-GS (glycine/serine). The ability to report transport activity was tested in liquid yeast cell cultures. Exchanges of cpEGFP with cpsfGFP in AmTrac-LE, -LS and -GS were well tolerated, as demonstrated by the growth complementation assay (Figure 5b). However, the resulting sfAmTrac-LE, -LS and -GS sensors were brighter (~10-fold, 24-fold and 36-fold, respectively) relative to AmTrac-LE (Supplementary Figure 16). The FI decrease in response to ammonium addition was ~40% for sfAmTrac-LE and –LS and 25% for sfAmTrac-GS, as compared to 37% of AmTrac-LE (Supplementary Figure 16).

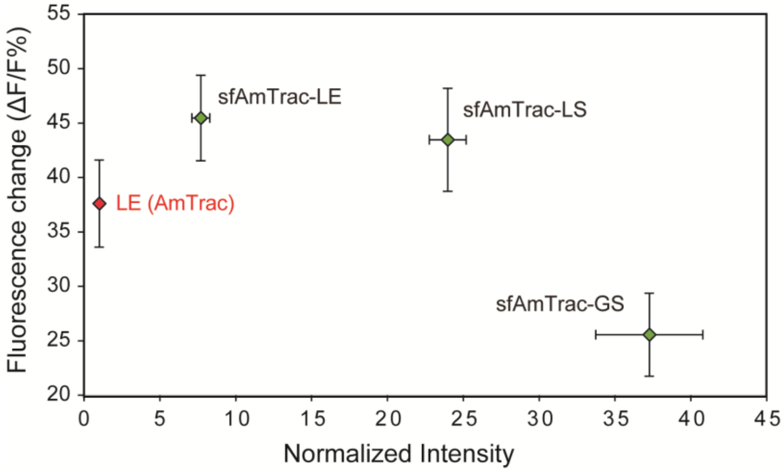

Supplementary Figure 16. Fluorescence change as response to 1 mM NH_4Cl versus basal FI (λ_{exc} 480 nm; λ_{em} 510 nm). Comparison of AmTrac-LE (in red)[2] with the cpsfGFP-based sfAmTrac variants, containing the left flanking peptides LE, LS or GS (green) (mean ± s.d., n = 3).

The sfAmTrac series served as basis to further test for potential effects of the F138I and L255I suppressor mutations, AtAMT1;3-F138I and -L255I recognition domains were generated in the sfAmTrac-LS and –GS sensors. Ammonium addition triggered a FI change of ~40% and ~20% for AmTrac-LS and –GS, respectively. However, the presence of either F138I or L255I increased the FI change to ~50% (AmTrac-LS-F138I or –L255I) and ~40% (AmTrac-GS-F138I or –L255I) in the flanking sequence variants. Thus, each individual point mutation increased the FI response up to 2-fold (Figure 5c; Supplementary Figure 17a). Analysis of the fluorescence intensity change (ΔF/F) in response to ammonium titration demonstrated similar ammonium affinities ($K_{0.5}$) compared to sfAmTrac-LS and –GS sensors (Supplementary Figure 17b; Supplementary Table 6).

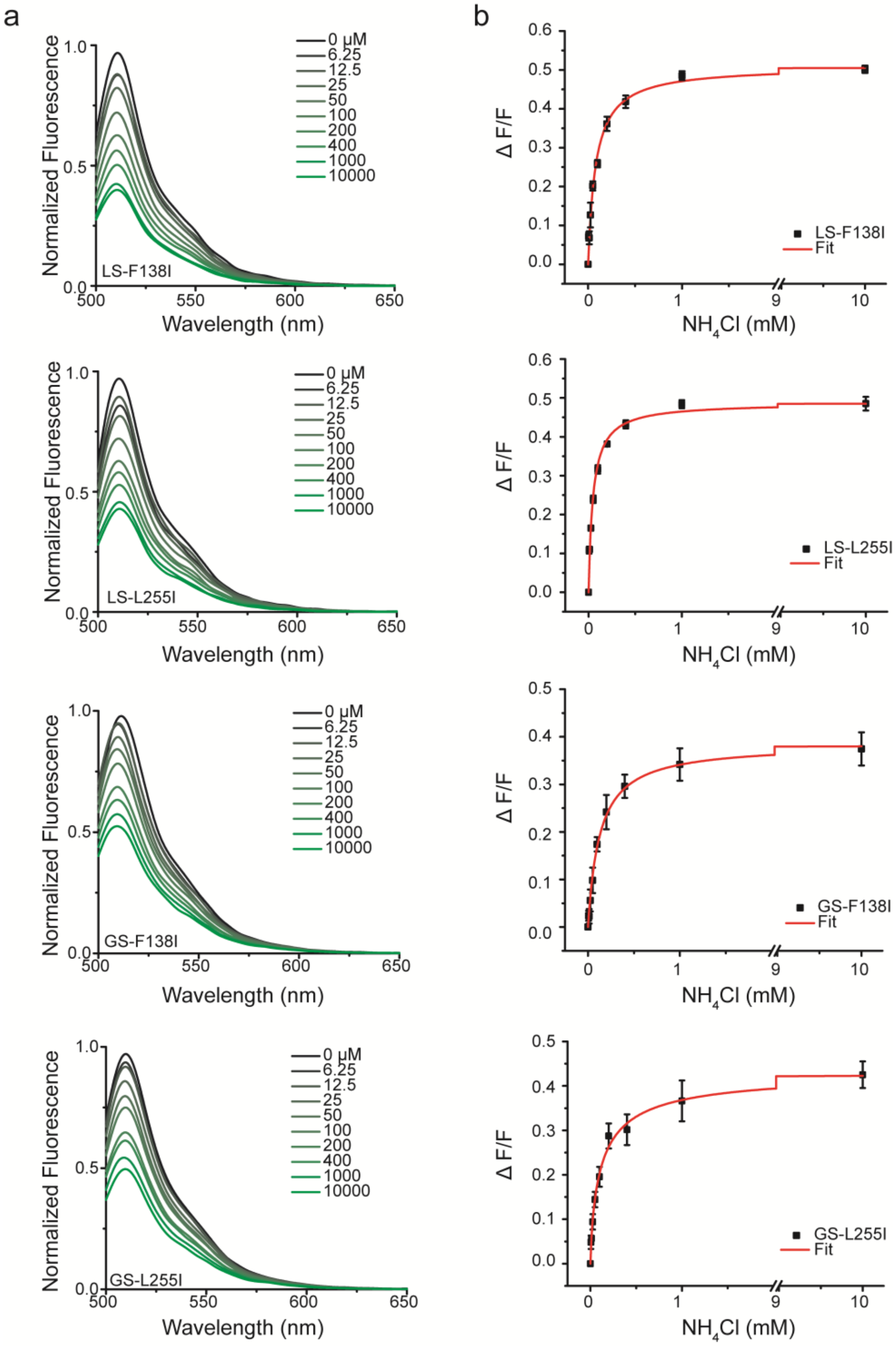

Supplementary Figure 17. Titration of sfAmTrac-LS and -GS by F138I or L255I suppressor mutations. (a) Steady-state emission spectra with λ_{exc} 440 nm after treatment with indicated NH_4Cl concentrations (0 - 10 mM) and normalized to water-treated control. (b) Corresponding titration curve of the fluorescence response ΔF/F (F = FI_{510nm}; black square) and the Hill fit (red line) (mean ± s.e.m.; *n* = 3). Affinities for ammonium transport derived from Hill plot are listed in Supplementary Table 6.

Supplementary Table 6 Affinities for ammonium transport of sfAmTrac set

**Sensor**	**$K_{0.5}$ (µM)**
sfAmTrac-LS-F138I	81.1 ± 7.1
sfAmTrac-LS-L255I	48.1 ± 3.8
sfAmTrac-GS-F138I	127.9 ± 20.6
sfAmTrac-GS-L255I	96.99 ± 19.1
sfAmTrac-LS	58.3 ± 15.1
sfAmTrac-GS	51.8 ± 4.4

Affinities were derived from Hill plot in Supplementary Figure 17b compared to non-mutated sfAmTrac-LS and –GS (mean ± s.e.m.; n = 4 for sfAmTrac-LS, n = 5 for sfAmTrac-GS, n = 3 for other sensors listed).

In summary, the exchange of cpEGFP for cpsfGFP in AmTrac resulted in increased FI up to 36-fold compared to the original AmTrac-LE. The individual suppressor mutations F138I and L255I improved the sensor response by 2-fold. Thus, the generation of sfAmTrac yielded a series of ammonium transport activity sensors that superseded the quality of the original AmTrac-LE.

Supplementary References

1. Chu, J. *et al.* A bright cyan-excitable orange fluorescent protein facilitates dual-emission microscopy and enhances bioluminescence imaging in vivo. *Nat. Biotechnol.* (2016). doi:10.1038/nbt.3550
2. De Michele, R. *et al.* Fluorescent sensors reporting the activity of ammonium transceptors in live cells. *eLife* **2,** e00800 (2013).
3. Ward, W. W., Prentice, H. J., Roth, A. F., Cody, C. W. & Reeves, S. C. Spectral Perturbations of the Aequorea Green-Fluorescent Protein. *Photochem. Photobiol.* **35,** 803–808 (1982).
4. Akerboom, J. *et al.* Genetically encoded calcium indicators for multi-color neural activity imaging and combination with optogenetics. *Front. Mol. Neurosci.* **6,** 2 (2013).
5. Chen, T.-W. *et al.* Ultrasensitive fluorescent proteins for imaging neuronal activity. *Nature* **499,** 295–300 (2013).
6. Barnett, L. M., Hughes, T. E. & Drobizhev, M. Deciphering the molecular mechanism responsible for GCaMP6m's Ca2+-dependent change in fluorescence. *PLOS ONE* **12,** e0170934 (2017).
7. Bizzarri, R. *et al.* Green Fluorescent Protein Ground States: The Influence of a Second Protonation Site near the Chromophore,. *Biochemistry* **46,** 5494–5504 (2007).
8. Scharnagl, C., Raupp-Kossmann, R. & Fischer, S. F. Molecular Basis for pH Sensitivity and Proton Transfer in Green Fluorescent Protein: Protonation and Conformational Substates from Electrostatic Calculations. *Biophys. J.* **77,** 1839–1857 (1999).
9. Oltrogge, L. M., Wang, Q. & Boxer, S. G. Ground-State Proton Transfer Kinetics in Green Fluorescent Protein. *Biochemistry* **53,** 5947–5957 (2014).

10. Chattoraj, M., King, B. A., Bublitz, G. U. & Boxer, S. G. Ultra-fast excited state dynamics in green fluorescent protein: multiple states and proton transfer. *Proc. Natl. Acad. Sci.* **93,** 8362–8367 (1996).
